# Unmanipulated native fat exposed to high-energy diet, but not autologous grafted fat by itself, may lead to overexpression of Ki67 and PAI-1

**DOI:** 10.1186/s40064-015-1061-0

**Published:** 2015-06-19

**Authors:** Francisco Claro, Joseane Morari, Luciana R Moreira, Luís O Z Sarian, Glauce A Pinto, Licio A Velloso, Aarão M Pinto-Neto

**Affiliations:** Department of Gynecology and Obstetrics at School of Medical Sciences, State University of Campinas (UNICAMP), R. Alexander Fleming, 101, Campinas, SP 13083-881 Brazil; Santa Cruz Plastic Surgery Institute (ICPSC), São Paulo, SP Brazil; Laboratory of Cell Signaling, Obesity and Comorbidities Research Center, State University of Campinas (UNICAMP), Campinas, SP Brazil; Laboratory of Specialized Pathology, School of Medical Sciences, State University of Campinas (UNICAMP), Campinas, SP Brazil

**Keywords:** Fat grafting, Breast cancer, Lipofilling, PAI-1, Ki67, CD68

## Abstract

**Background:**

Although its unclear oncological risk, which led to more than 20 years of prohibition of its use, fat grafting to the breast is widely used nowadays even for aesthetic purposes. Thus, we proposed an experimental model in rats to analyze the inflammatory activity, cellular proliferation and levels of Plasminogen Activator Inhibitor (PAI-1) in grafted fat, and in native fat exposed to high-energy diet in order to study the oncological potential of fat tissue.

**Methods:**

Samples of grafted fat of rats on regular-energy diet were compared with paired samples of native fat from the same rat on regular-energy diet and on high-energy diet in a different time. Analysis involved microscopic comparisons using hematoxylin-eosin staining, immunohistochemistry with anti-CD68-labelled macrophages, and gene expression of Ki-67 and PAI-1.

**Results:**

Hematoxylin-eosin staining analyses did not find any atypical cellular infiltration or unusual tissue types in the samples of grafted fat. The inflammatory status, assessed through immunohistochemical identification of CD68-labelled macrophages, was similar among samples of native fat and grafted fat of rat on regular-energy diet and of native fat of rats on high-energy diet. Real-time PCR revealed that high-energy diet, but not fat grafting, leads to proliferative status on adipose tissue (overexpression of ki-67, *p* = 0.046) and raised its PAI-1 levels, *p* < 0.001.

**Conclusion:**

While the native adipose tissue overexpressed PAI-1 and KI67 when exposed to high-energy diet, the grafted fat by itself was unable to induce cellular proliferation, chronic inflammatory activity and/or elevation of PAI-1 levels.

## Background

In 1895, Czerny described the first breast reconstruction, which was performed with adipose tissue, using a large lipoma from the dorsal flank to fill defects resulted from the excision of a breast benign lesion (Claro et al. 2012). Since then, the adipose tissue (as fat grafting or pedicle flaps from great omentum or subcutaneous tissue) became often used for breast reconstruction (Claro et al. 2012, 2015; Abbott and White 1986; Calderoli and Keiling 1985; Góes 2010; Illouz 1988; Kiricuta 1963). Its popularity though, appeared after the advent of liposuction in the 1970s, when the aspirated fat harvested from many body areas could be reinjected to the breast (Claro et al. 2012). So, the autologous fat grafting began to be used for aesthetic purposes as well, once it is performed using a non-immunogenic substitute/filler, through a versatile and inexpensive procedure obtained usually without donor site morbidity (Claro et al. 2012).

However, it was suggested that adipose tissue might represent an oncological risk for breast cancer, what led the American Society of Plastic Surgeons to prohibit its use to the female breast in 1987 (Claro et al. 2012; ASPRS 1987; Chalmers and Newing 1986). Most of this theory was raised after some studies have correlated the higher potential of breast cancer in obese people (Carter and Church 2009, 2012). Long since great focus to adipose tissue in obesity has been given, such as its comparisons between the omentum and subcutaneous tissue in many disorders like diabetes and cancer (Harman-Boehm et al. 2007; Rodbell 1964; Tam et al. 2012; Weisberg et al. 2003). The most notable hypothesis related to adipocytes and breast cancer is based on the inflammatory potential of these cells, which release adipokines that may lead to chronic inflammation and cell proliferation (once the risk for metabolic disorders and breast cancer seems to be higher in obese people) (Claro et al. 2012, 2015; Chalmers and Newing 1986; Carter and Church 2009, 2012). However, this hypothesis has not been sustained for the procedure of lipofilling to the breast in clinical practice.

Thereupon, the lack of evidence for lipofilling and breast cancer almost 20 years after its prohibition lead to the publication of some case series reporting breast reconstruction with fat grafting in early 2000’s, with good results and without report of higher cancer recurrence than other well established reconstruction procedures (Claro et al. 2012; Góes and Macedo 2006). This demanded in 2009, a review of the prohibition imposed by the American Society of Plastic Surgeons that, owing to lack of evidence, failed to prohibit the use of autologous adipose tissue to the breast, although they do not recommend it (Gutowski and Force 2009).

Since then, many cases series and reviews remain failing to demonstrate higher recurrence rate of breast cancer among women treated with fat grafting (Claro et al. 2012, 2015). So, great effort in experimental field has been made in order to analyze the oncological potential of fat cells to the breast. Some laboratorial studies have focused on adipocytes in vitro, while others have analyzed fat cells from people due to the great difficulty in established an effective experimental model in vivo (Carter and Church 2009, 2012; Baglioni et al. 2009; Baumert et al. 2007; Lin et al. 2001; Wyckoff et al. 2004). Most of these studies even compared fat cells from different fat compartments and/or from people in different conditions, such as obesity and non-obesity. Regarding these issues, great focus to Plasminogen Activator Inhibitor-1 (PAI-1) complex has been taken, because it is a protein found in high concentration in adipose tissue of obese people and/or of breasts with cancer and so, can be used as a marker for them (Carter and Church 2009, 2012; Harman-Boehm et al. 2007; Baglioni et al. 2009; Andreasen et al. 2000; Bianchi et al. 1995; Binder et al. 2002; Cojocaru et al. 2012; Condeelis and Pollard 2006; Dhanasekaran et al. 2012; Di Gregorio et al. 2005; Gomes-Giacoia et al. 2013; Goswami et al. 2005; Gutierrez et al. 2000; Lin et al. 2002; Sumiyoshi et al. 1991). Some of these studies were able to demonstrate the oncological potential of the adipocytes, primary in obese subjects. However, this potential remains uncertain for the procedure of lipofilling to the breast by itself, and was not observed in clinical practice. One believed reason for this, raised by us, is that the breast bed, even after a complete resection of mammary tissue, remains with a large amount of native adipose tissue. So, for locally oncological potential, the adipokines released from transposed/grafted fat cells would be the same of those released from native adipocytes that are already surrounding any remaining mammary glandular cell. And systemically, considering that the donor fat cells are from the same patient that will receive them, the serum concentration of any pro-oncological factor resulting from these adipocytes will remain the same after the procedure.

Moreover, beyond the adipocyte, some theories raised doubts regarding the procedure by itself, due to the great angiogenic potential of the adipose tissue observed in some clinical and experimental studies (Dhanasekaran et al. 2012; Figueiredo et al. 2010; Goldsmith et al. 1967; Liebermann-Meffert 2000; Morison 1903; O’Shaughnessy 1937; Oloumi et al. 2006; Williams and White 1991), that may lead to chronic inflammation and cell proliferation to the microenvironment of lipofilling host site and its surroundings. Thus, there are some issues that must be considered when studying fat grafting: (1) the unpredictable amount of grafted fat present in the sample extracted from the host site, because the long-term graft retention is inaccurate and its absorption can vary from less than 10% to more than 90%; (2) as fat graft is composed not only by adipocytes but also by many other cells and components present in fat tissue, the whole elements of the adipose tissue though, must be analyzed after fat grafting; (3) the grafted tissue must be analyzed in vivo in order to study its behavior in the host site and of its surroundings and; (4) the subjects must be genetically similar and, as controlled as possible, in relation to its diet intake and lifestyle.

Thus, an experimental model was proposed taken into account all the challenges reported above in order to analyze the carcinogenic potential of autologous fat grafting procedure focusing on its main suggested threats to the host site (higher concentration of PAI-1, chronic inflammation activity and higher proliferation rate). We also aimed to compare the findings for this potential resulted from the procedure by itself to those represented by the unmanipulated native fat tissue when exposed to high-energy diet.

## Methods

### Study design and sample size

A comparative, paired and controlled experimental study was conducted using eight female Sprague–Dawley rats (*Rattus norvegicus*) from the same dam; obtained at 56-day-old from the Central Reproduction Centre at the State University of Campinas (UNICAMP), Campinas-SP, Brazil. The Sprague–Dawley rat was chosen because it is a breed prone to develop breast cancer when exposed to some risk factors (Russo and Russo 1996; Russo et al. 1983; Shan et al. 2004). Considering that a variable (PAI-1 levels) was used as positive and a negative control for the same tissue of the same rat in different times, a sample size of three rats was defined as enough. However, considering possible exclusions during the evolution of the study, the sample size was set to eight rats.

**Figure 1:**
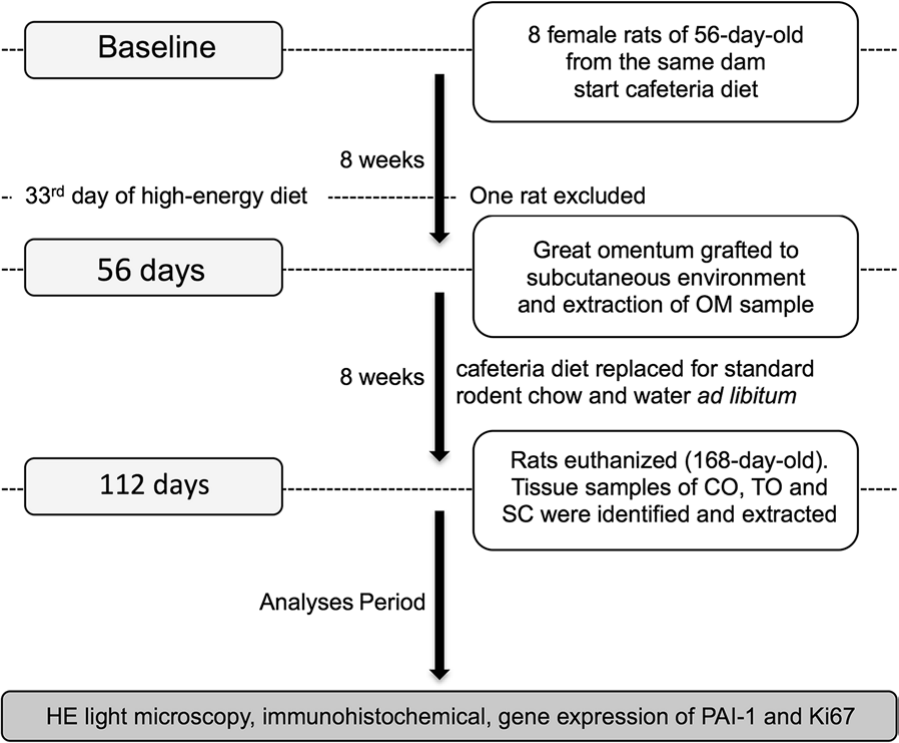
Flowchart overview of the study. *OM* unmanipulated native fat tissue of omentum of rats on cafeteria diet regimen (high-energy diet), *CO* native fat tissue of omentum of rats in regular-energy diet, used as post-operative control, *TO* grafted fat tissue of omentum of rats in regular-energy diet, *SC* fragment of unmanipulated native subcutaneous adipose tissue from the left iliac fossa used as control for fat tissue similarity, *HE* hematoxylin-eosin.

The study followed the ARRIVE guidelines (Kilkenny et al. 2010) and was approved by the UNICAMP Committee for Ethics in Animal Research (protocol no. 2210-1). The rats remained in the animal-breeding center at the Institute of Biology, UNICAMP from the beginning of the fattening period to the collection of the material for analysis. The rats were housed in isolated boxes that were cleaned daily under a 12-h/day light cycle at a constant temperature of 22 ± 2°C. An overview of the study is summarized in Figure 1.

### Experimental model proposed and adipose tissue chosen

In order to avoid bias related to the unpredictable amount of grafted fat present in the sample extracted from the host site, an experimental model was proposed using a pedicle fat graft of great omentum, instead of free fat graft from subcutaneous tissue, based on deep study of the literature, and discussions among researchers from some departments of biological institute and medical school at UNICAMP. Although they are from different embryological origin, the adipose tissue from omentum and subcutaneous tissue have the same cellular composition, with similar regenerative and physiological function, and even the same amount of stem cells able to differentiate into any mesenchymal tissue (Cancello et al. 2006; Dicker et al. 2009; Toyoda et al. 2009). A difference identified between these two adipose tissues is the greater metabolic and inflammatory potential of the great omentum if compared to the subcutaneous tissue. These issues are stated as oncogenic risk for breast cancer, what became this model of fat grafting using fat tissue of omentum, instead of subcutaneous, even more sensitive. Thus, in order to avoid bias related to this and false-positive results, a control of native subcutaneous fat of the same rat was used as control for fat tissue similarity.

### Dietary regimen and composition of diet

Cafeteria diet was used for 56 days, until the time of great omentum grafting (rats of 112-day-old), so we were able to analyze the carcinogenic potential of high-energy foods consumed by humans in adipose tissue. After the great omentum translocation, the cafeteria diet was replaced for standard rodent chow and water ad libitum for more 56 days (until rats’ age of 168-day-old).

The cafeteria diet consists of replacement of water for soft drinks ad libitum (Coca-cola®) and of standard rodent chow for a pellet made of 37.5% standard rodent chow, 25% peanuts, 25% chocolate, and 12.5% cookies, offered together with wafer, snacks, cakes, and biscuits (4.41 kcal/g, 43.1% from carbohydrates; 12.1% from proteins, and 46.9% from fats). The standard rodent chow diet Nuvilab CR-1 (Nuvital, Brazil) has 2.63 kcal/g (Vanzela et al. 2010). The cafeteria diet was used at the beginning of the study when the rats were younger and so, less prone to bias due to other metabolic disorders related to age. With the cafeteria diet we were able to confirm that the changes in results were only due to the replacement of the diet on the same rat (from high-energy to regular-energy) and so, analyze the influence of diet over the fat tissue and its oncological potential.

### Surgical procedure

The rats underwent general anesthesia (intraperitoneally) at 112-day-old. The omentum grafting process was performed through a midline skin incision of approximately 4 cm. Subcutaneous dissection of the entire right hemi-abdomen followed by laparotomy with a 4-cm incision at abdominal midline. The great omentum was divided into three parts (three samples for the study): (1) one was made with 20 mm^2^ of the left pedicle flap in the left gastroepiploic branch and remained in the abdominal cavity (fat tissue control—CO); (2) a portion of the right flap (also 20 mm^2^) based on the right gastroepiploic branch was grafted into the subcutaneous layer in the upper right abdomen and fixed to the abdominal wall at its ends with 5.0 nylon (transposed omentum—TO); (3) the remaining central portion of omentum was used to analyze the influence of high-energy diet over fat tissue (omentum on high-energy diet—OM). The abdominal wall was closed with running sutures of 5.0 nylon except the upper 0.3 cm, which was left open for the passage of the translocated omental pedicle. Finally, the skin was closed with running sutures of 5.0 nylon.

The rats were euthanized at 8 weeks post-operatively (168-day-old). Three tissue samples were identified and extracted: native fat tissue of omentum (CO), the grated fat tissue (TO); and a fragment of unmanipulated native subcutaneous adipose tissue (SC) from the left iliac fossa (an area with abundant adipose tissue distant from the manipulated surgical site, thus free from any postoperative inflammatory process).

### Histological preparation and analysis

Each sample was immediately identified and introduced into a vial containing 10% buffered formalin. The tissue samples were processed in the pathology laboratory. Sections of 4 μm of each sample were placed on glass slides, dehydrated and stained with hematoxylin-eosin (HE). The evaluation was performed by a single pathologist blinded to the origin of the tissues (with the samples identified only by numbers and letters) with light microscopy, in order to identify the presence of cell populations that differ from the normal cells in the adipose tissue.

### Immunohistochemistry

Immunohistochemistry using monoclonal anti-mouse anti-CD68 antibodies (clone Kp-1, Advance, Dako, Glostrup, Denmark) was used to identify the macrophage concentrations in each tissue type (Weisberg, Harman-Boehm) according to the manufacturer’s instructions. A single pathologist, blinded to the origin of each sample, performed the immunohistochemical analysis. The number of anti-CD68 stained macrophages was counted in ten different randomly chosen areas in each processed slide at 40× magnification for each tissue sample. A cell was considered positive when the morphological aspects of a macrophage were observed with a marked cytoplasm outside the vascular lumen (Cancello et al. 2006; Aron-Wisnewsky et al. 2009).

### Real-time PCR

Total mRNA extraction was performed using TRIzol^®^ Reagent protocol (Life Technologies, #15596018). Ki67 and PAI-1 mRNA expression were measured in all groups by Real Time PCR (ABI Prism 7500—Applied Biosystems). The primer Ki67 (Rn.PT.58.8428180.g) was obtained from Integrated DNA Technology (IDT), and PAI-1 (Rn01481341_m1) were purchased from Applied Biosystems. GAPDH (#4352339E—Applied Biosystems) was used as endogenous control. Each PCR contained 40 ng of reverse-transcribed RNA, 0.25 μl of each specific primer, Taqman Universal master mix (#4369016—Applied Biosystems), and RNase free water to a 10 μl final volume. Real-time data were analyzed using the Sequence Detector System 7500 (Applied Biosystems).

### Statistical analyses

The normal distribution of the data was assessed using the Kolmogorov–Smirnov and Shapiro–Wilk tests. As the data were normally distributed, there were used paired t-test and analysis of variance (ANOVA, one way, and repeated measures) and Tukey test (post hoc) paired t-test for statistical comparisons. The values are given as mean ± standard deviation (SD) and the significance level was 5%. The software used for the analysis was SPSS version 20 for MAC (IBM; Armonk, NY—EUA).

## Results

One of the rats died on the 33rd day of high-energy diet, at 89-day-old, before the procedure of adipose tissue grafting. Then, seven rats remained and were used for analyses in this study. A complete healing of the surgical wound with full hair growth at the site was observed at time of samples extraction (rats’ age of 168-day-old). All samples revealed viable flaps under microscopic analysis (without necrosis). No atypical cellular infiltration or unusual tissue types were observed in the samples of grafted fat.

### High-energy diet or fat grafting did not influence the inflammatory activity involving CD68 macrophages in fat tissue

**Figure 2:**
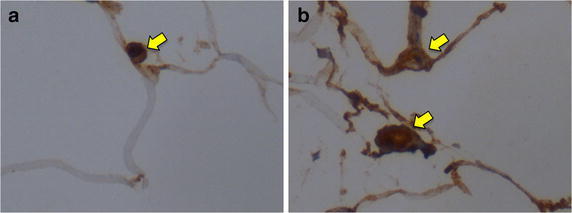
CD68-labelled macrophages (*yellow arrow*) in **a** fat tissue of rats fed with high-energy food (OM) and in **b** grafted fat of rats in regular-energy diet (TO) (magnification ×400).

**Figure 3:**
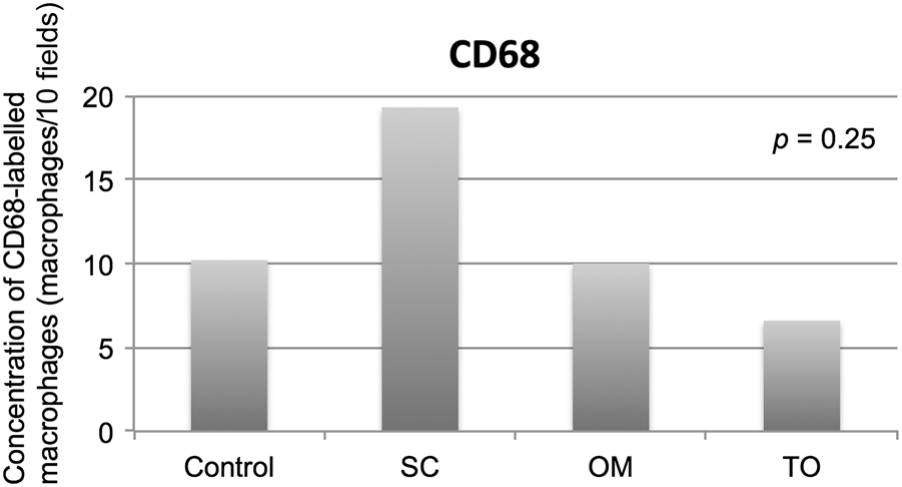
Immunohistochemical analyses of the concentration of CD68-labelled macrophages (macrophages/10 fields) per animal in unmanipulated native fat tissue of omentum of rats fed with high-energy food (OM), in native fat tissue of omentum of rats in regular-energy diet (CO), in fat of omentum of rats in regular-energy diet grafted to subcutaneous environment (TO) and in the fat tissue of unmanipulated native subcutaneous adipose tissue from the left iliac fossa (SC).

Immunohistochemical analyses of the average concentration of CD68-labelled macrophages (Figure 2) in unmanipulated native fat tissue samples of rats fed with high-energy food (OM) was 10.00 ± 4.02 macrophages/field in 10 fields per animal; the average among samples of those fed with standard rodent chow was 10.17 ± 4.17 in control native fat (CO); 6.60 ± 1.75 in grafted fat (TO), and 19.33 ± 5.60 in the subcutaneous fat used as control for fat tissue similarity (SC). What represents that the inflammatory activity involving CD68 macrophages among all the adipose tissue samples were similar *p* = 0.246 (CI 95% 7.03–16.17), Figure 3.

### The high-energy diet represents higher proliferation rate in the adipose tissue environment

**Figure 4:**
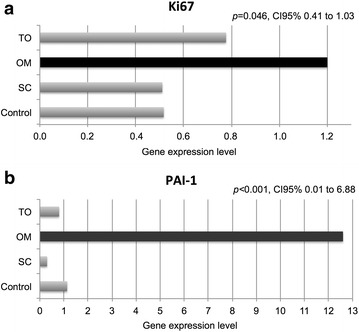
Gene expression level of **a** Ki67 and **b** PAI-1. *OM* unmanipulated native fat tissue of omentum of rats on cafeteria diet regimen (high-energy diet), *CO* native fat tissue of omentum of rats in regular-energy diet, used as post-operative control, *TO* grafted fat tissue of omentum of rats in regular-energy diet, *SC* fragment of unmanipulated native subcutaneous adipose tissue from the left iliac fossa used as control for fat tissue similarity.

The gene expression level of Ki67 (Figure 4a) in the CO samples was 0.52 ± 0.03. A similar pattern was observed in the SC (0.51 ± 0.11) however; the level of 1.20 ± 0.00 was significantly higher in the OM (ANOVA OM vs. CO and SC: *p* = 0.046, CI 95% 0.41–1.03).

### The high-energy diet, but not fat grafting procedure, leads to elevation of PAI-1 levels in adipose tissue

Gene expression of PAI-1 (Figure 4b) revealed that its level in the OM of 12.63 ± 3.07 is significantly higher than those identified in CO (1.13 ± 0.32), SC (0.29 ± 0.12) and TO (3.44 ± 1.58), *p* < 0.001, CI 95% 0.01–6.88. The ANOVA test shows similar low levels of PAI-1 gene expression (*p* = 0.70) among samples of rats fed with standard rodent chow (CO, TO and SC).

## Discussion

Our results showed that the grafted adipose tissue and its surroundings did not expressed inflammatory activity mediated by macrophages, higher cellular proliferation rate nor higher levels of PAI-1. However, high-energy diet leads to higher cellular proliferative rate in the unmanipulated native adipose tissue (analyzed through Ki67) and higher levels of PAI-1.

It is known that the adipose tissue provides a source of stem cells (i.e., cells that are capable of differentiating into other cell types of the same embryonic origin) (Baglioni et al. 2009; Baumert et al. 2007; Dhanasekaran et al. 2012; Oloumi et al. 2006; Kobayashi et al. 2006) and exhibits great angiogenic potential (Figueiredo et al. 2010; Oloumi et al. 2006) for restoring ischemic tissues and actinic lesions (Claro et al. 2012; Illouz 1988; Goldsmith et al. 1967; Liebermann-Meffert 2000; Morison 1903; Williams and White 1991). The movement of this tissue from its primary site to a different environment might expose it to metaplasia. In the present study this theory was not proved, once the histological results using HE did not reveal the presence of cell metaplasia in the TO.

The CD68 is a membrane glycoprotein type 1, strongly expressed by tissue macrophages, present in inflammatory events of vascular and adipose tissues, usually related to fat metabolism. These macrophages have been cited as present in high concentration in adipose tissue of patients with plurimetabolic syndrome and/or insulin-resistance (Di Gregorio et al. 2005), in atherosclerotic plaques (Cojocaru et al. 2012), and in breasts with cancer (with unfavorable impact on disease invasion and progression) (Lin et al. 2001; Wyckoff et al. 2004; Condeelis and Pollard 2006; Goswami et al. 2005; Lin et al. 2002; Piras et al. 2005; Soeda et al. 2008; Offersen et al. 2003). However, according to our findings, no connection was identified in literature between the level of CD68-labelled macrophages and obesity in patients without any metabolic disorder (Tam et al. 2012; Di Gregorio et al. 2005). What represents that those macrophages levels in a tissue depend of inflammatory events and pathological chemotaxis from sick adipocyte mediators but not from healthy adipose tissue. In this study, the CD68 antigen was used to identify the chemotaxis potential of adipose tissue to attract CD68-labelled macrophages, which may represent a risk for breast cancer and/or its unfavorable evolution (Lin et al. 2001; Wyckoff et al. 2004; Condeelis and Pollard 2006; Goswami et al. 2005; Lin et al. 2002; Piras et al. 2005; Soeda et al. 2008).

Di Gregorio et al. (2005) have shown that the CD68 expression is also higher at the stromal vascular fraction of the adipose tissue than its adipocyte fraction. Considering that the angiogenesis is intense just after adipose tissue grafting or translocation (Figueiredo et al. 2010), what represents one among many theories for its potential for breast cancer, a chronic higher concentration of CD68-labelled macrophage was expected. However, this data was not found in this study and the CD68 expression was similar in all adipose tissue samples. Therefore, adipose tissue grafting does not seem to generate chronic inflammatory activity involving CD68 macrophages that may lead to a carcinogenic potential in its host microenvironment.

Ki67 is a nuclear protein present in proliferating cells, but absent in resting cells. What means that ki67 is associated with cell proliferation (Gerdes et al. 1984; Pathmanathan and Balleine 2013; Rahmanzadeh et al. 2007). Nowadays, Ki67 scoring is used as a prognostic factor for early breast cancer and even as a predictor of its treatment efficacy (Pathmanathan and Balleine 2013; Urruticoechea et al. 2005; Bullwinkel et al. 2006; Luporsi et al. 2012; Inwald et al. 2013; Nishimura et al. 2010). The gene expression of Ki67 was used in order to evaluate the proliferation rate of adipose tissue from different body compartments, as well as its behavior on high-energy diet and after its translocation to a different environment. The possibility of transposed fat creating a proliferative status in the host microenvironment is considered mostly due to the knowledge that the adipose tissue promotes angiogenesis, deeply explored in the great omentum experiments (Baumert et al. 2007; Figueiredo et al. 2010; Goldsmith et al. 1967; O’Shaughnessy 1937; Oloumi et al. 2006), what theoretically may represent another threat for breast cancer. In this study, the adipose tissue per se, did not show any change in its proliferative rate according to its origin site or after its translocation to a different environment. However, a proliferative status was observed on samples of unmanipulated native fat exposed to a high-energy dietary regimen.

Plasminogen Activator Inhibitor 1 is a single-chain glycoprotein that acts as the primary regulator of plasminogen activation in vivo. It is secreted by several cells and participates in tissue repair processes (Binder et al. 2002; Gomes-Giacoia et al. 2013; Sumiyoshi et al. 1991; Jankun et al. 1993). High PAI-1 level correlates with obesity, hyperinsulinemia, hyperglycemia, and hypertriglyceridemia (Carter and Church 2009; Binder et al. 2002). Some oncological conditions show high level of PAI-1 as well. Its role in breast cancer has been widely studied, where it is involved in the decrease of apoptotic activity, degradation of extracellular matrix during tumor growth, invasion, and metastasis. High levels of PAI-1 in breast cancer thus, is linked to the poor prognosis of disease progression (Carter and Church 2009, 2012; Andreasen et al. 2000; Bianchi et al. 1995; Gomes-Giacoia et al. 2013; Gutierrez et al. 2000; Sumiyoshi et al. 1991; Offersen et al. 2003; Jankun et al. 1993; Foekens et al. 2000). Our study found high levels of PAI-1 only on unmanipulated adipose tissue samples of rats fed with high-energy food, which posteriorly became low in the same rats after change of their diet regimen. Carter and Church (2012) demonstrated that the native mature fat cells of breast seems to represent a greater threat to breast cancer than fat cells from other regions and, even higher than immature adipocytes or stem cells. In addition, fat cells are widely present in our body and there is no evidence that a fat grafting procedure by itself would bring additional oncological risk to a region that is already surrounded by adipose tissue.

## Conclusion

We found in this study in rats that, while the unmanipulated native adipose tissue overexpressed PAI-1 and KI67 when exposed to high-energy diet, the grafted fat by itself was unable to induce cellular proliferation, chronic inflammatory activity and/or elevation of PAI-1 levels. This findings highlight that fat grafting procedure alone does not seem to change the oncological potential of its host microenvironment, however more experimental studies focusing on the fat grafting behavior in vivo must be made in order to confirm this issue.
